# 

**Published:** 1889-08

**Authors:** 


### BOOK NOTICES.

We are under obligations to the several authors for copies of the following essays and treatises :

Life Sketch of Hon. John A. Collins.

THE VACCINATION DILEMMA, ETC., by E. Haughton, M. D., B. A., London.

THE HOME MAKER.—Edited by Marion Harland and published monthly at 19 W. 22d st., New York City, is one of the very best family magazines obtainable. As such we commend it to our readers. Single copies 20 cents.

TABLE TALK is another family monthly of real merit. Its seasonable menus are of great use to the household. Published by Table Talk Co., 406 Race st., Philadelphia.

THE CENTURY MAGAZINE.—Back numbers of this foremost monthly, containing the Lincoln and Siberian papers, can be had of the publishers. Address, The Century Co., New York City.

THE SATELLITE.—Quarterly of the Medical Science for May, is at hand. Charles E. Sojous, M. D., editor. The medical and surgical information contained in these quarterlies is of too great value to the profession to be lost sight of. No practitioner can afford to do without it. F. A. Davis, publisher, 1231 Filbert st., Philadelphia ; 45 E. 12th st., New York.

We congratulate the *World's Advance Thought* and the *Universal Republic*, yoke-fellows in good works, on their new dress. A vast improvement over the old.

